# Adaptive control of airway pressure during the expectoration process in a cough assist system

**DOI:** 10.3389/fbioe.2024.1477886

**Published:** 2024-10-23

**Authors:** Liangsong Lu, Yixuan Wang, Guolang Shen, Minghua Du

**Affiliations:** ^1^ Beihang University, Beijing, China; ^2^ Ceramic University, Jiangxi, China; ^3^ Institute of Stomatology, First Medical Center, Chinese PLA General Hospital, Beijing, China

**Keywords:** cough assist, MI-E, airway pressure, adaptive control, clinical safety

## Abstract

Existing Mechanical Insufflation-Exsufflation (MI-E) devices often overlook the impact of cough airflow pressure on mucus clearance, particularly lacking in control over airway pressure during the expiratory phase, which can lead to airway collapse and other types of airway damage. This study optimizes the design of cough assist system and explores the effectiveness of PID and adaptive control methods in regulating airway pressure. The adaptive control method compensates for hose pressure drop by online estimation of the ventilatory hose characteristics. It achieves precise tracking of target pressure and ensures the generation of peak flow rates effective for mucus clearance, even in the absence of known patient lung physiological states and unknown hose leakage parameters. Through a series of comparative experiments, this paper confirms the significant advantages of adaptive control in reducing oscillations and overshoot, capable of more stable and precise airway pressure adjustments. This improved control strategy not only enhances clinical safety but also significantly improves therapeutic outcomes and reduces the risk of complications. The findings indicate that the revamped cough assist system, employing an adaptive control strategy, can effectively prevent airway damage during assisted coughing, offering a safer and more effective sputum clearance solution for critically ill patients with expectoration disorders.

## Introduction

Cough represents an expedited reflex mechanism in the human body, the purpose of which is to expel foreign bodies or particles from the respiratory tract, thereby safeguarding the respiratory system. (Dubuis et al., 2014; Laghi et al., 2017; Zhang et al., 2017). The process is initiated by a deep inhalation, introducing a substantial volume of air into the lungs. Subsequently, the laryngeal muscles proceed to close the glottis, while the abdominal and thoracic muscles contract, generating elevated pressure within the pulmonary system. Ultimately, the glottis opens abruptly, and the expiratory muscles contract forcefully to release the air (Fink, 2007). However, for patients with neuromuscular diseases (NMDs) or disorders like emphysema that impair the function of respiratory muscles, effective clearance of secretions can be compromised due to weak or impaired cough (Burle et al., 2018). Mechanical ventilation is a common intervention for the intraoperative and postoperative management of critical patients (Kwong et al., 2019). In the intensive care unit (ICU), mechanically ventilated patients may suffer from impaired cough due to the impediment of glottic closure by endotracheal tubes and the frequent use of sedatives and muscle relaxants that are concomitant with mechanical ventilation. This impairment of coughing can lead to the accumulation of airway secretions, potentially resulting in respiratory failure, inadequate ventilation, tracheobronchitis, pneumonia, or other complications associated with secretion retention (de Camillis Márcio Luiz et al., 2018). Consequently, assistive cough devices are essential for the clearance of secretions in patients with compromised cough ability.

In the early 1950s, Barach et al. introduced a physical method called the “mechanical coughing machine” to simulate some of the mechanisms of human cough (Bickerman et al., 1952; Barach et al., 1952). Clinical observations of patients with poliomyelitis revealed that the maximal expiratory flow rate averaged 1.6 L/s, which is 145% of that produced by the most forceful natural cough. Based on the advancements in non-invasive ventilation, assistive coughing devices were subsequently refined, employing blowers to provide positive pressure for lung inflation and negative pressure for rapid lung deflation, later termed Mechanical Insufflation-Exsufflation (MI-E). MI-E delivers up to 60 cm H2O of positive and negative pressure to generate large peak cough flows, applying high shear forces to the secretions and propelling them into the oral cavity. Barach et al. conducted several clinical studies on peak cough flow based on MI-E (Bach, 1993; Bach, 1995; Bach et al., 1997). With patient cooperation, peak cough flow rates could reach 6 L/s or higher. Recently, MI-E has found numerous applications in secretion clearance for patients with Chronic Obstructive Pulmonary Disease (COPD), NMDs, and other types of respiratory muscle dysfunction. Additionally, MI-E has been utilized for mechanically ventilated patients in the ICU. As one of the most effective methods of simulating natural cough, MI-E can propel bronchial secretions to the central airways, which cannot be cleared by tracheal suctioning (Terzi et al., 2018). However, due to the reduced consciousness and lack of cooperation in most patients, CPFR values are typically less than 3 L/s, diminishing the efficacy of airway secretion clearance. Concurrently, the generation of substantial negative pressure during the exsufflation phase may lead to airway collapse.

The airflow volume and pressure generated during a cough are key determinants of the effectiveness of sputum excretion. (Ren et al., 2022). Airflow volume dictates the amount of secretion or foreign matter that can be carried and moved by the cough. Theoretically, a greater volume increases the efficiency of clearing secretions or foreign matter from the respiratory tract. Increased airflow volume, and consequently higher velocities, assist in disrupting the cohesion of mucus, detaching it from the airway surfaces, and expelling it from the body. A higher airflow volume enhances the ability to penetrate into the distal airways, playing a crucial role in clearing the lower respiratory tract. (Yi et al., 2021). Furthermore, the pressure of airflow during a cough is equally critical to its effectiveness. Sufficient airflow pressure is capable of opening airways that are partially obstructed due to mucus or constriction, rendering the cough more effective. Elevated pressure aids in overcoming the adhesive force of mucus, thereby facilitating its movement from the airway walls. If the pressure is sustained over time, this contributes to the continuity of the cough, ensuring a more thorough clearance of the airways. (Ashley Piccone, 2022).

Recent studies have extensively investigated the impact of airflow rates on the efficacy of coughing. Parameters such as Cough Expiratory Volume (CEV), Peak Velocity Time (PVT), and Cough Peak Flow Rate (CPFR) are utilized to quantify and describe the outcomes of coughing actions (Mahajan et al., 1994; Singh et al., 1995; Vansciver et al., 2011; Ren et al., 2021). Devices based on the Mechanical Insufflation-Exsufflation (MI-E) principle also aim to improve expectoration efficiency by leveraging these cough airflow parameters. However, existing MI-E cough assist systems have not considered the influence of cough airflow pressure on therapeutic effectiveness. Additionally, these devices lack effective solutions to control airway pressure during the expiratory phase, where significant negative pressure can lead to airway collapse.

Current research on respiratory airflow pressure is extensively conducted in the context of mechanical ventilation. Researchers explore various control strategies to enhance mechanical ventilators. In reference (Borrello, 2005), the modeling and control techniques of mechanical ventilation are outlined. References (Van De Wouw et al., 2018) and (Hunnekens et al., 2020) propose variable gain control, aimed at achieving pressure tracking while reducing overshoot in patient flow and preventing mis-triggering. This article demonstrates a notable reduction in patient flow overshoot. However, some overshoot still occurs, and the control strategy uses patient flow, which is often unavailable. Reference (Borrello, 2001) applies an adaptive feedback control method that estimates the patient model and uses this model to adaptively adjust the controller to achieve the desired closed-loop transfer function. Theoretically, this approach is very effective, but obtaining an accurate patient model is complex in practice. Moreover, in (Borrello, 2001), hose resistance is overlooked, yet the pressure drop caused by the hose cannot be ignored for large airflow caused by large lungs and/or leaks. Furthermore, funnel-based control (Pomprapa et al., 2015) applied in mechanical ventilation shows limited gains in tracking performance. Model-based control approaches used in (Scheel et al., 2017) and model predictive control methods applied in (Li and Haddad, 2012) require precise patient parameters, which are often unavailable in practice. Additionally, Iterative Learning Control (Scheel et al., 2015) applied to mechanical ventilation has shown significant improvements in tracking performance. However, this method is limited to the repetitive sequence of set points and initial conditions. Consequently, the performance of the Iterative Learning Control framework proposed in (Scheel et al., 2015) diminishes when patients breathe autonomously. However, research on such airway pressure control is rare in cough assist systems that require stronger expiratory airflow.

Therefore, this study proposes an improved design of the MI-E cough assist system. By employing adaptive control, the device effectively regulates airway pressure during the assisted coughing process facilitated by the cough assist system, offering a novel solution to enhance safety and prevent airway damage during expectoration in critically ill patients with expectoration disorders.

## Methods

### Principles of the cough assist system

The cough assist system is capable of simulating the entire coughing process, comprising both inhalation and exhalation phases found in human respiratory mechanics. Considering the inability of patients with impaired cough reflex to forcefully inhale and exhale, the system employs a brushless direct current (DC) micro-turbine fan to supply air to the lungs, thereby expanding lung capacity to mimic the inhalation process observed in healthy individuals. Concurrently, a combination of a vacuum pump and a vacuum tank is used to generate a near-vacuum negative pressure state, facilitating the suction required to simulate cough airflow.

**FIGURE 1:**
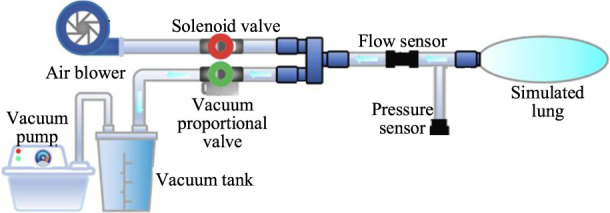
Working principle and system components of the cough assist system.

During the inflation process, an electromagnetic valve (VX234, SMC Ltd., Tokyo, Japan) opens, while a vacuum proportional valve (ITV0090, SMC Ltd., Tokyo, Japan) remains closed. The micro-turbine fan supplies air to the simulated lungs at a predefined pressure to mimic the inhalation phase of coughing. Simultaneously, the vacuum pump extracts air from the storage tank, creating a near-vacuum state. A flow sensor (FS6122, Siargo Ltd., Santa Clara, USA) is employed to measure the cough airflow, and a pressure sensor (XGZP6847, CFSensor, Wuhu, China) monitors the airway pressure. When the airway pressure reaches the predefined inflation value, the electromagnetic valve closes, concluding the inflation process. At this juncture, a significant negative pressure difference is established between the simulated lungs and the vacuum tank. By adjusting the vacuum proportional valve, air is rapidly expelled from the simulated lungs into the vacuum tank, simulating the exhalation phase of a natural cough. The control of the vacuum proportional valve allows for the adjustment of the simulated cough airway pressure, enhancing safety during the expectoration process by preventing airway damage. The working principle and system composition of the cough assist machine are illustrated in Figure 1.

### Patient-hose dynamics

**FIGURE 2:**
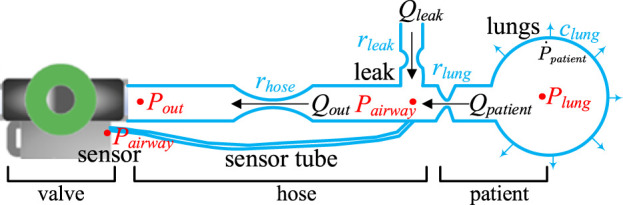
Mathematical model of the cough assist system.

Before discussing the mathematical model that describes the interaction between the cough assist machine and the patient during the expectoration process, it is essential to define the physical quantities involved, with reference to the mathematical model of the cough assist system shown in Figure 2 (Reinders et al., 2021). Firstly, all pressures are defined relative to the ambient pressure, thus $P_{amb}=0$ . The vacuum proportional valve is responsible for opening the connection between the vacuum tank and the hose, generating the cough airflow. This negative pressure suction results in flow through the hose, which encounters resistance $r_{hose}$ . Additionally, a pressure sensor measures the airway pressure in front of the patient’s mouth, $P_{airway}$ . Potential airflow leakage at the connection between the patient and the mask is modeled using a leakage resistance $r_{leak}$ . The lung model employs a linear single-compartment model as described in reference (Ashley Piccone, 2022), which includes the compliance $c_{lungs}$ and resistance $r_{lungs}$ of the lungs. Notably, all physical parameters, namely, $r_{hose}$ , $r_{leak}$ , $r_{lungs}$ , and $c_{lungs}$ , are positive values. Figure 2 includes ${\dot{P}}_{patient}$ representing the patient’s spontaneous respiratory effort, which is considered an exogenous disturbance to the lung pressure caused by the patient’s breathing effort or autonomous coughing action.

Utilizing the parameters and model discussed previously, a mathematical model between the patient and the hose has been derived. This model delineates the relationships between the pressure at the outlet of the vacuum proportional valve ( $P_{out}$ ) the disturbance from the patient’s spontaneous breathing ( ${\dot{P}}_{patient}$ ), the state variable representing lung pressure ( $P_{lungs}$ ), and the outputs airway pressure ( $P_{airway}$ ), and output flow rate ( $Q_{out}$ ). The output flow rate $Q_{out}$ , the patient’s flow rate $Q_{patient}$ , and the leakage flow rate $Q_{leak}$ are interrelated, and are expressed using Equation 1.

$$Q_{patient}=Q_{out}-Q_{leak}$$ (1)

The model employs a linear resistance framework, which provides reasonable accuracy for typical ventilation flows. Linear resistances $r_{hose}$ , $r_{leak}$ , and $r_{lungs}$ are utilized to express the relationships between pressure and flow rates, as delineated in Equations 2–5.

$$Q_{out}=\frac{P_{airway}-P_{out}}{r_{hose}}$$ (2)

$$Q_{leak}=\frac{P_{airway}}{r_{leak}}$$ (3)

$$Q_{patient}=\frac{P_{lungs}-P_{airway}}{r_{lungs}}$$ (4)

$$P_{airway}=\frac{r_{leak}r_{hose}P_{lungs}+r_{leak}r_{lungs}P_{out}}{r_{leak}r_{lungs}+r_{leak}r_{hose}-r_{hose}r_{lungs}}$$ (5)

The dynamics of lung function are encapsulated in Equation 6.

$$P_{lungs}\left(t\right)=\frac{1}{c_{lungs}}\int_{0}^{t}Q_{patient}dt+P_{patient}\left(t\right)+P_{lungs}\left(0\right)$$ (6)

In the model, $P_{patient}\left(t\right)$ , which varies over time, represents the patient’s own physiological activity. This activity is simulated as an unknown disturbance in lung pressure caused by the patient’s respiratory or coughing efforts, such as contractions of the diaphragm and/or abdominal muscles. Additionally, $P_{lungs}\left(0\right)$ denotes the initial lung pressure, excluding any effort by the patient. The derivative of lung pressure with respect to time is expressed in Equation 7.

$${\dot{P}}_{lungs}=\frac{1}{c_{lungs}}\times Q_{patient}+{\dot{P}}_{patient}$$ (7)

Combining Equations 3, 7, the dynamics of the lung are represented by Equation 8.

$${\dot{P}}_{lungs}=\frac{1}{c_{lungs}}\times \left(\frac{P_{lungs}-P_{airways}}{r_{lungs}}\right)+{\dot{P}}_{patient}$$ (8)

By substituting Equation 5 into Equation 8, this research derive the differential equation that governs lung dynamics.

$$\begin{array}{l} {\dot{P}}_{lungs}=\frac{\left(r_{leak}r_{lungs}-r_{hose}r_{lungs}\right)P_{lungs}-r_{leak}r_{lung}P_{out}}{r_{lungs}c_{lungs}\left(r_{leak}r_{lungs}+r_{leak}r_{hose}-r_{hose}r_{lungs}\right)}\\ +{\dot{P}}_{patient} \end{array}$$ (9)

According to Equation 9, the dynamics of the patient-hose system can be formulated as a linear state-space system with inputs. This model uses $P_{out}$ as the input, $P_{airway}$ and $Q_{patient}$ as the outputs, and $P_{lungs}$ as the state variable. Additionally, ${\dot{P}}_{patient}$ acts as a disturbance within the system Equations 11–14.

$$\begin{array}{l} {\dot{P}}_{lungs}=A_{hose}P_{lungs}+B_{hose}P_{out}+{\dot{P}}_{patient}\\ \left[\begin{array}{l} P_{airway}\\ Q_{patient} \end{array}\right]=C_{hose}P_{lungs}+D_{hose}P_{out} \end{array}$$ (10)

with

$$A_{hose}=\frac{r_{leak}r_{lungs}-r_{hose}r_{lungs}}{r_{lungs}c_{lungs}\left(r_{leak}r_{lungs}+r_{leak}r_{hose}-r_{hose}r_{lungs}\right)}$$ (11)

$$B_{hose}=\frac{-r_{leak}r_{lungs}}{r_{lungs}c_{lungs}\left(r_{leak}r_{lungs}+r_{leak}r_{hose}-r_{hose}r_{lungs}\right)}$$ (12)

$$C_{hose}=\left[\begin{array}{l} \frac{r_{leak}r_{hose}}{r_{leak}r_{lungs}+r_{leak}r_{hose}-r_{hose}r_{lungs}}\\ \frac{r_{leak}r_{lungs}-r_{hose}r_{lungs}}{r_{lungs}\left(r_{leak}r_{lungs}+r_{leak}r_{hose}-r_{hose}r_{lungs}\right)} \end{array}\right]$$ (13)

$$D_{hose}=\left[\begin{array}{l} \frac{r_{leak}r_{lungs}}{r_{leak}r_{lungs}+r_{leak}r_{hose}-r_{hose}r_{lungs}}\\ \frac{-r_{leak}r_{lungs}}{r_{lungs}\left(r_{leak}r_{lungs}+r_{leak}r_{hose}-r_{hose}r_{lungs}\right)} \end{array}\right]$$ (14)

Given that all resistances and compliances in the system are positive, $A_{hose}$ is negative, indicating that the patient-hose system is inherently asymptotically stable. Notably, ${\dot{P}}_{patient}$ is considered an exogenous disturbance; however, in practical terms, it includes dynamics associated with the patient’s breathing or autonomous coughing behavior.

### Statement of control objectives

Figure 2 Presents a schematic diagram of the expectoration process within the cough assist system, highlighting its most critical components. This system operates by utilizing a vacuum proportional valve to regulate the connection between the vacuum tank and the hose, thereby generating a cough airflow through negative pressure to assist the patient in expectorating. One end of the hose is connected to the vacuum proportional valve, while the other end is attached to a mask that interfaces with the patient’s mouth. The airflow exits the mouth, travelling through the hose towards the vacuum tank. Airflow leakage near the patient’s mouth along the hose is also a source of airflow, as illustrated in Figure 2.

**FIGURE 3:**
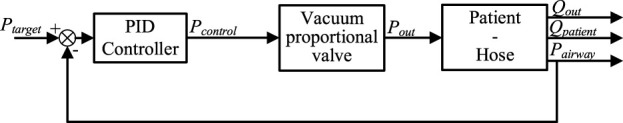
Pid closed-loop system diagram for the expectorator process.

In engineering applications, a PID (Proportional, Integral, Derivative) controller is commonly utilized to address new control demands. Similarly, in the cough assist system driven by a vacuum proportional valve, a PID controller can be employed to achieve the new control objective of regulating pneumatic pressure. Employing a PID controller to manage this system results in a closed-loop system, as depicted in Figure 3.

Within this closed-loop framework, the airway pressure ( $P_{airway}$ ) is the variable that requires control, with $P_{target}$ being the target pressure that the system aims to track. The overall control objective is to minimize the tracking error, defined as follows Equation 15:

$$e=P_{target}-P_{airway}$$ (15)

or ideally let it converge to zero asymptotically.

However, the PID controller must be robustly adjusted to accommodate significant variations in the controlled object. For patients with different conditions, variations in lung compliance and airway resistance are common. Consequently, a PID controller may not achieve accurate tracking for all patients considered.

**FIGURE 4:**
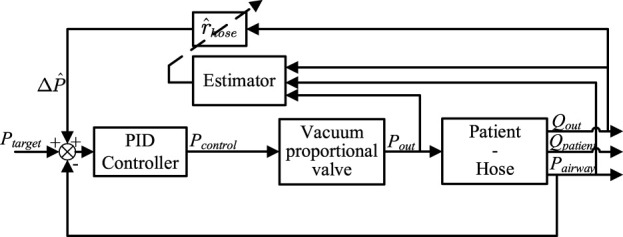
Control diagram of the closed-loop system with feedforward estimation.

Therefore, it is considered advantageous to introduce a feedforward element ∆P to predict and compensate for the pressure drop between the patient and the hose. If ∆P can be accurately estimated, it would significantly enhance the control precision and robustness of the control system. The system control diagram incorporating the feedforward element is illustrated in Figure 4.

It is noteworthy that predicting pressure drops within the system is not a trivial task due to various factors.

1) The status of the lungs in different patients is fundamentally unknown. Although the pressure targets are known *a priori*, the flow emanating from the lungs depends on lung resistance and compliance, which are variables that remain unknown. Consequently, the flow through the hose and the pressure drop P across it are also unknown.
2) The characteristics of the hose system used are also unknown. Consequently, the pressure drop along the hose remains an unknown variable.
3) During the assisted expectoration process, leakage around the mask may occur, which is unpredictable and thus leads to previously unknown pressure drops.
4) Patients may exhibit varying degrees of spontaneous coughing or breathing activities, which generate airflow and result in pressure drops; these dynamics are also unpredictable.

Therefore, feedforward prediction and compensation of pressure drops need to account for multiple variable factors.

### Control strategy

**FIGURE 5:**
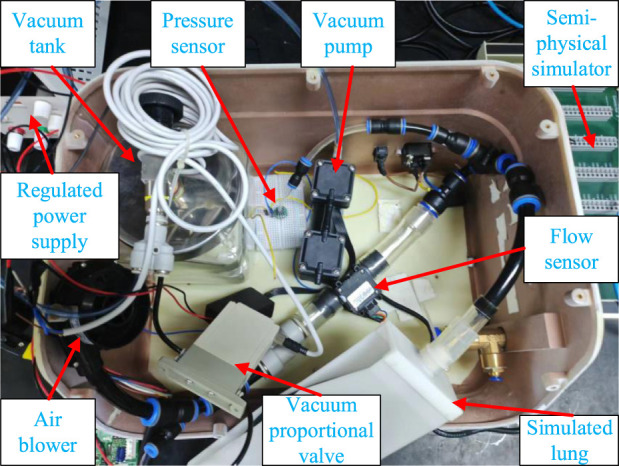
Experimental setup display.

A novel adaptive control method is proposed, employing an online Recursive Least Squares (RLS) estimator to automatically estimate hose impedance during the expectoration process as shown in Figure 5. The proposed strategy utilizes airway pressure to update the estimator.

Another advantage of this control strategy is that it compensates for the hose pressure drop P using the measured flow rate $Q_{out}$ from the vacuum proportional valve outlet. The hose pressure drop P depends on the flow through the hose, which equals the flow rate $Q_{out}$ from the vacuum proportional valve. Therefore, precise compensation of the pressure drop based on the measured flow rate can achieve target pressure tracking that is independent of leaks, patient lung dynamics, and the patient’s own physiological actions.

Subsequently, a state-space description of the closed-loop dynamics with a constant ${\hat{r}}_{hose}$ estimation is derived, as illustrated in Figure 5. In the diagram, the desired $P_{control}$ is equal to $P_{out}$ . Using $P_{out}=\hat{P}+P_{target}$ and Equation 10 results in

$${\dot{P}}_{lungs}=A_{hose}P_{lungs}+B_{hose}\left(P_{target}+∆\hat{P}\right)+{\dot{P}}_{patient}$$ (16)

From Figure 5, it can be determined that the estimated pressure drop $\hat{P}$ is given by $\hat{P}={\hat{r}}_{hose}Q_{out}$ . The estimated value of the pressure drop $\hat{P}$ can be expressed by Equation 17.

$$\begin{array}{l} ∆\hat{P}={\hat{r}}_{hose}Q_{out}\\ ={\hat{r}}_{hose}\left(Q_{patient}+Q_{leak}\right)\\ ={\hat{r}}_{hose}\left(c_{lungs}\left({\dot{P}}_{lungs}-{\dot{P}}_{patient}\right)+\frac{P_{airway}}{r_{leak}}\right) \end{array}$$ (17)

Note that $P_{control}=\hat{P}+P_{target}$ and Equation 17 essentially establish the proposed feedback law, which is designed to compensate for the pressure drop in the hose system. Substituting Equation 8 into Equation 17 yields Equation 18.

$$\begin{array}{l} ∆\hat{P}={\hat{r}}_{hose}(c_{lungs}\left(1+\frac{r_{lungs}}{r_{leak}}\right)\left({\dot{P}}_{lungs}-{\dot{P}}_{patient}\right)\\ +\frac{P_{lungs}}{r_{leak}}) \end{array}$$ (18)

For notational purposes, the combined variable Equation 19

$$r\left(e_{r}\right)=e_{r}\left(r_{leak}+r_{lungs}\right)+r_{leak}r_{lungs}$$ (19)

Equation 20 defines the estimation error $e_{r}$ .

$$e_{r}=r_{hose}-{\hat{r}}_{hose}$$ (20)

Substituting Equation 18 into Equation 16 results in Equation 21.

$${\dot{P}}_{lungs}=\frac{-r_{leak}-e_{r}}{c_{lungs}r\left(e_{r}\right)}P_{lungs}+\frac{r_{leak}}{c_{lungs}r\left(e_{r}\right)}P_{target}+{\dot{P}}_{patient}$$ (21)

Thus, the state space of the new closed-loop system can be represented by Equation 22.

$$\begin{array}{l} {\dot{P}}_{lungs}=A\left(e_{r}\right)P_{lungs}+B\left(e_{r}\right)P_{target}+{\dot{P}}_{patient}\\ \left[\begin{array}{l} P_{airway}\\ Q_{patient}\\ Q_{out} \end{array}\right]=C\left(e_{r}\right)P_{lungs}+D\left(e_{r}\right)P_{target} \end{array}$$ (22)

with

$$\begin{array}{l} A\left(e_{r}\right)=\frac{-r_{leak}-e_{r}}{c_{lungs}r\left(e_{r}\right)},B\left(e_{r}\right)=\frac{r_{leak}}{c_{lungs}r\left(e_{r}\right)}\\ C\left(e_{r}\right)={\left[1-\frac{\left(r_{leak}+e_{r}\right)r_{lungs}}{r\left(e_{r}\right)}\frac{-r_{leak}-e_{r}}{r\left(e_{r}\right)}\frac{-r_{leak}}{r\left(e_{r}\right)}\right]}^{T}\\ D\left(e_{r}\right)={\left[\frac{r_{leak}r_{lungs}}{r\left(e_{r}\right)}\frac{r_{leak}}{r\left(e_{r}\right)}\frac{r_{leak}+r_{lungs}}{r\left(e_{r}\right)}\right]}^{T} \end{array}$$ (23)

It is noteworthy that the dynamics described in Equation 22 are actually nonlinear due to the system matrix’s dependence on the estimation error $e_{r}$ . Subsequently, analysis of the system using a constant least squares estimation error $e_{r}$ is conducted. Specifically, this study requires obtaining linear dynamics when $e_{r}=0$ to understand the behavior of the closed-loop system under conditions where resistance estimation is available and pressure compensation is utilized. This analysis is achieved through the transfer function of the linear system with a constant estimation error. Through the transfer function, robust performance characteristics of the closed-loop system were obtained.

Using the system dynamics in Equations 22, 23, we can calculate the transfer function from the inputs $P_{target}$ and ${\dot{P}}_{patient}$ to the output $P_{airway}$ . Consequently, the closed-loop system is reformulated as follows Equations 24–26:

$$\begin{array}{l} {\dot{P}}_{lungs}=\bar{A}P_{lungs}+\bar{B}u\\ P_{airway}=\bar{C}P_{lungs}+\bar{D}u \end{array}$$ (24)

Where the input vector $u={\left[P_{target}{\dot{P}}_{patient}\right]}^{T}$

$$\begin{array}{l} \bar{A}=A\left(e_{r}\right),\bar{B}=\left[B\left(e_{r}\right)1\right]\\ \bar{C}=C_{1}\left(e_{r}\right),\bar{D}=\left[D_{1}\left(e_{r}\right)0\right] \end{array}$$ (25)

Using this form of the closed-loop system, the transfer function from u to $P_{airway}$ can be derived.

$$\frac{P_{airway}\left(s\right)}{u\left(s\right)}=\bar{C}{\left(s-\bar{A}\right)}^{-1}\bar{B}+\bar{D}$$ (26)

Using this, the expression for $P_{airway}$ can be obtained.

$$P_{airway}\left(s\right)=P_{1}P_{target}\left(s\right)+P_{2}{\dot{P}}_{patient}\left(s\right)$$ (27)

with

$$P_{1}=\frac{r_{leak}+c_{lungs}r_{leak}r_{lungs}s}{r_{leak}+c_{lungs}r_{leak}r_{lungs}s+e_{r}\left(1+c_{lungs}\left(r_{leak}+r_{lungs}\right)s\right)}$$

and

$$P_{1}=\frac{c_{lungs}e_{r}r_{leak}}{r_{leak}+c_{lungs}r_{leak}r_{lungs}s+e_{r}\left(1+c_{lungs}\left(r_{leak}+r_{lungs}\right)s\right)}$$

Assuming that the airway resistance value is accurately estimated, meaning the estimation error $e_{r}=0$ the transfer function from $P_{target}$ to $P_{airway}$ , denoted as $P_{1}$ in Equation 27, can be derived. Additionally, $P_{2}$ represents the transfer function from ${\dot{P}}_{patient}$ to $P_{airway}$ . It can be seen that the airway pressure does not depend on the patient dynamics or the exogenous disturbances caused by the patient’s own physiological activities, denoted as ${\dot{P}}_{patient}$ . This independence is a highly desirable characteristic for the controlled system.

Subsequently, an RLS (Recursive Least Squares) estimator with an exponential forgetting factor β is employed to automatically estimate the value of $r_{hose}$ during the expectoration process, as illustrated in Figure 5 (Ioannou and Sun, 1996). In the estimation of airway resistance, because data from the distant past is considered less important than more recent data, an RLS algorithm featuring an exponential forgetting factor is utilized (Ioannou and Sun, 1996).

The RLS estimator with a forgetting factor is represented by Equations 28 and 29.

$${\dot{\hat{r}}}_{hose}=C\frac{∆P-{\hat{r}}_{hose}Q_{out}}{m^{2}}Q_{out}$$ (28)

$$\dot{C}=\beta C-C^{2}\frac{Q_{out}^{2}}{m^{2}}$$ (29)

Where $Q_{out}$ is the excitation variable, $C\left(t\right)$ is referred to as the covariance, and $\left(∆P-{\hat{r}}_{hose}Q_{out}\right)/\left(m^{2}\right)$ represents the normalized estimation error of the pressure drop, while $m^{2}$ is a constant normalization parameter. As $∆P=r_{hose}Q_{out}$ , $e_{r}\left(t\right)=r_{hose}-{\hat{r}}_{hose}\left(t\right)$ and $r_{hose}$ is constant, the dynamics of the least squares error can be represented by Equation 30.

$${\dot{e}}_{r}=-C\frac{Q_{out}^{2}}{m^{2}}e_{r}$$ (30)

The final closed-loop dynamics, incorporating the estimator and hose compensation controller, are given by Equations 22, 23, 29, 30.

Appropriate parameters β and $C\left(0\right)$ should be selected to enable rapid convergence of the system. Additionally, the constant normalization parameter $m^{2}$ is set to one to reduce the number of tuning parameters.

### Experimental setup

Experimental validation of the control effectiveness for regulating gas pressure is conducted using an experimental setup.

The cough assist system’s experimental platform, as shown in Figure 5, primarily consists of critical components such as a vacuum pump, a vacuum tank, a vacuum proportional valve, a flow sensor, a pressure sensor, fan, simulated lungs, as well as a power source and a Rapid Control Prototyping (RCP) system. To ensure real-time execution of test experiments, the RCP system using the Maple Technologies MT-1050 is employed for signal acquisition from the flow and pressure sensors and for outputting voltage signals. The proposed control strategy is implemented in the Simulink Desktop Real-Time software environment, and the necessary. a files are generated through the compiler options to achieve real-time closed-loop control.

The initial parameter settings during the experimental process are shown in Table 1.

**TABLE 1:** Initial parameter settings.

Parameter	Value	Unit
β	0.65	1/s
$C\left(0\right)$	2.5 × 10^−3^	$s/mL^{2}$
${\hat{r}}_{hose}\left(0\right)$	0	mbars/L
$m^{2}$	1	−

## Results

### Results of PID control adjustment of airway pressure during the Expectorator Process

Considering the lack of real-time pressure variation curves generated by cough airflow during a patient’s coughing process, this study opts to validate control effectiveness using square wave and sinusoidal pressure signals. Square and sinusoidal signals are two of the most fundamental and widely used signal types, capable of simulating behavioral patterns in various physical and physiological processes. Sinusoidal signals provide continuous, periodic variations, which are useful for testing the system’s performance under smoother pressure changes. This can emulate the periodic breathing patterns found in actual physiological environments. Square wave signals, with their distinct transition points, test the system’s response to rapid changes. How the system quickly adapts during the airway pressure drop phase is a crucial aspect for verifying the sensitivity and accuracy of the ventilator control system. Moreover, the sharp pressure changes of the square wave signal align with the normal operating conditions of cough assist systems generating negative pressure cough airflow, allowing for the measurement of peak flow rates to ensure effective mucus clearance.

**FIGURE 6:**
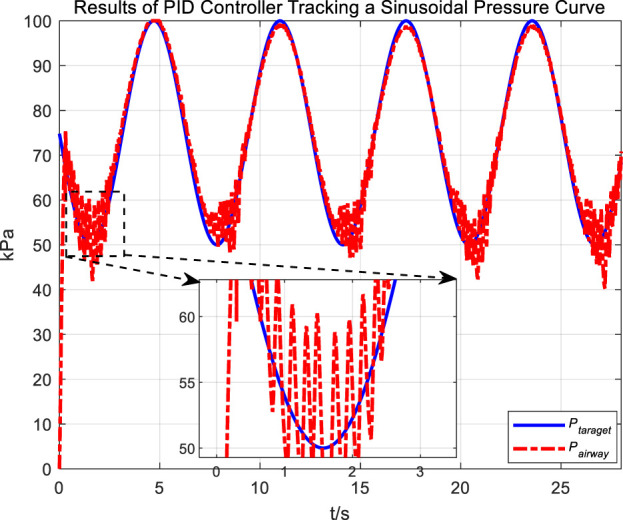
Results of PID controller tracking a sinusoidal pressure curve.

**FIGURE 7:**
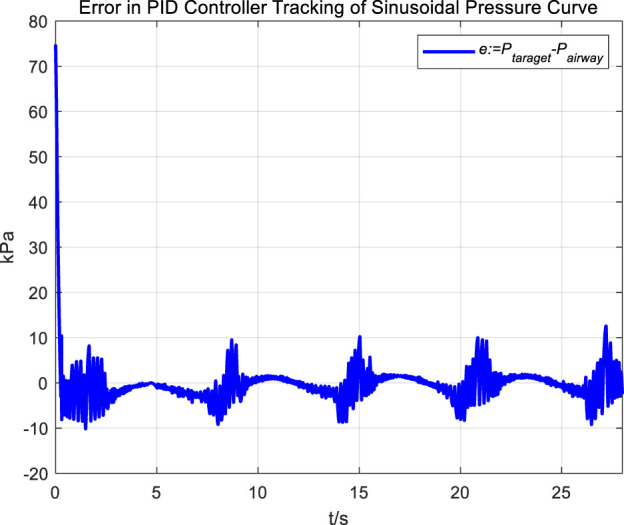
Error in PID controller tracking of sinusoidal pressure curve.

In the experiment, an initial attempt is made to adjust airway pressure using a PID controller, aiming for the airway pressure to track a target sinusoidal pressure curve. The tracking performance of the airway pressure curve under PID control and the corresponding tracking error are represented in Figures 6, 7, respectively.

The tracking results of a sinusoidal pressure curve using a PID controller revealed significant overshoot and oscillation in airway pressure regulation during the expectoration exhalation process, with errors reaching up to ±10 kPa.

The occurrence of these results can be explained by the following considerations. The airflow is generated by the vacuum generated by the opening and closing of the vacuum proportional valve, and the rapid change in flow rate, combined with the compressibility of the gas, leads to pressure fluctuations and oscillations. Additionally, there are leaks in the system; when air leaks, it causes changes in internal pressure. The PID controller attempts to correct the pressure changes caused by leaks. If the size or rate of the leak does not match the pressure compensation capability of the control system, these sudden changes in pressure and subsequent compensation can lead to pressure fluctuations and oscillations.

**FIGURE 8:**
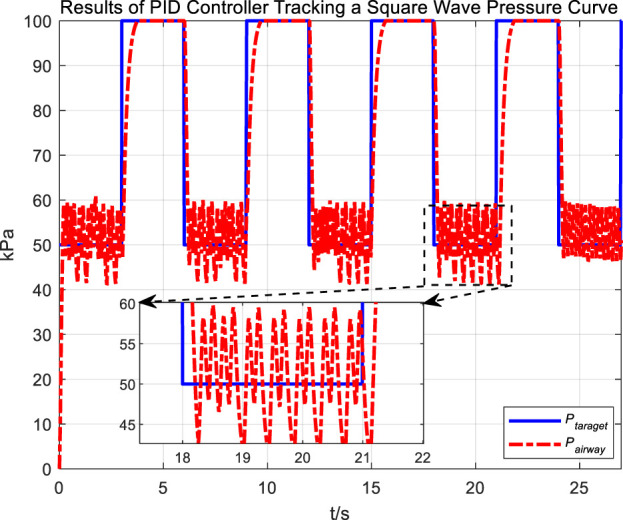
Results of PID controller tracking a square wave pressure curve.

**FIGURE 9:**
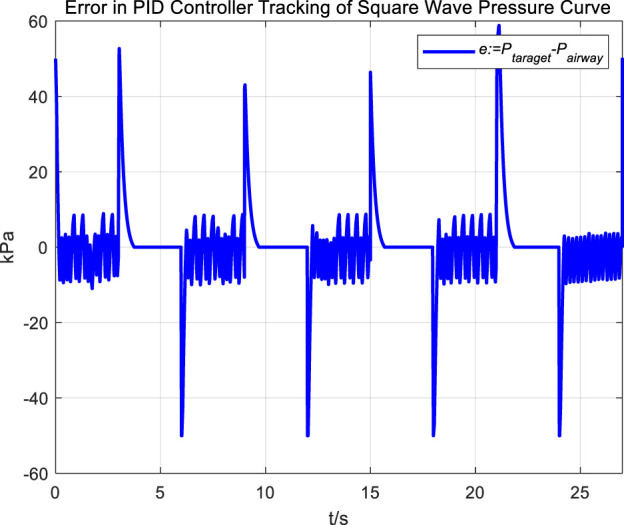
Error in PID controller tracking of square wave pressure curve.

Following the observation of airway pressure tracking a sinusoidal pressure curve under PID control, we examined the tracking performance of airway pressure against a square wave pressure curve under PID control. The tracking effectiveness and corresponding tracking error are depicted in Figures 8, 9, respectively.

Observations of the PID controller’s tracking results for the square wave pressure curve led to conclusions similar to those obtained from tracking the sinusoidal pressure signal. During the expectoration exhalation process, the regulation of airway pressure resulted in significant overshoot and oscillation, with errors reaching up to ±10 kPa. The reasons for the overshoot and oscillation are consistent with those previously discussed.

### Results of adaptive control adjustment of airway pressure during the Expectorator Process

This section of the results validates the feasibility and superiority of the adaptive control strategy proposed in this study for regulating airway pressure during the expectoration process.

**FIGURE 10:**
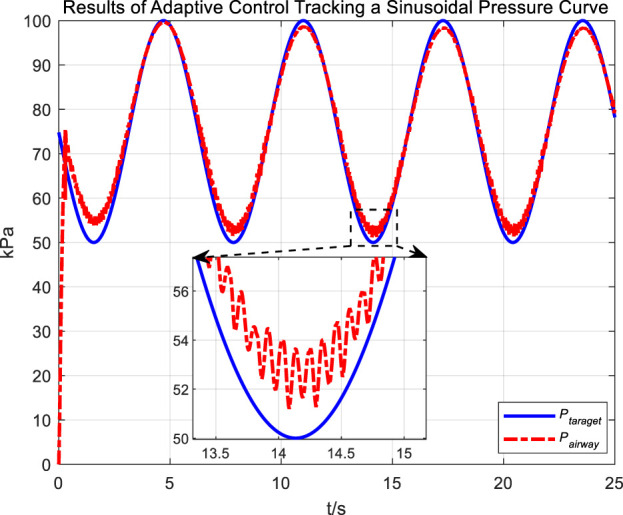
Results of adaptive control tracking a sinusoidal pressure curve.

**FIGURE 11:**
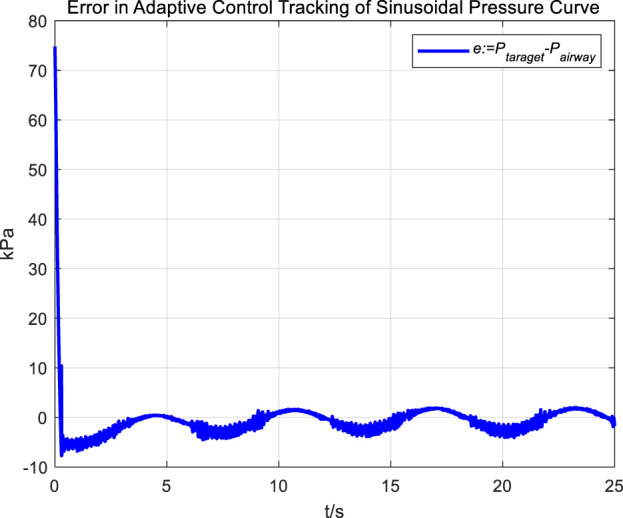
Error in adaptive control tracking of sinusoidal pressure curve.

**FIGURE 12:**
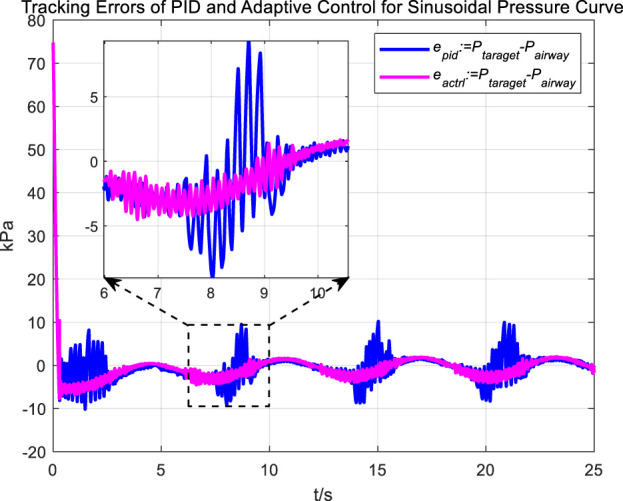
Tracking errors of PID and adaptive control for sinusoidal pressure curve tracking.

Similarly, we first obtained the results of airway pressure tracking a sinusoidal pressure signal under the adaptive control strategy. The tracking performance is displayed in Figures 10, 11. Comparing these results with those obtained under PID control, it is straightforward to observe the differences in tracking error variations for the sinusoidal pressure signal under the two control strategies, as shown in Figure 12.

Observations from the results indicate that under the adaptive control strategy, the regulation of airway pressure during the expectoration exhalation process has significantly improved. There is no overshoot, and although there is a steady-state error of approximately −3 kPa, oscillations have been reduced to ±1 kPa, a tenfold decrease. This effectively ensures comfort and safety in the airways during patient expectoration.

The significant improvement in control effectiveness with the adaptive control method is primarily due to its online estimation of ventilatory hose characteristics, which compensates for hose pressure drops under conditions of unknown hose leakage parameters.

**FIGURE 13:**
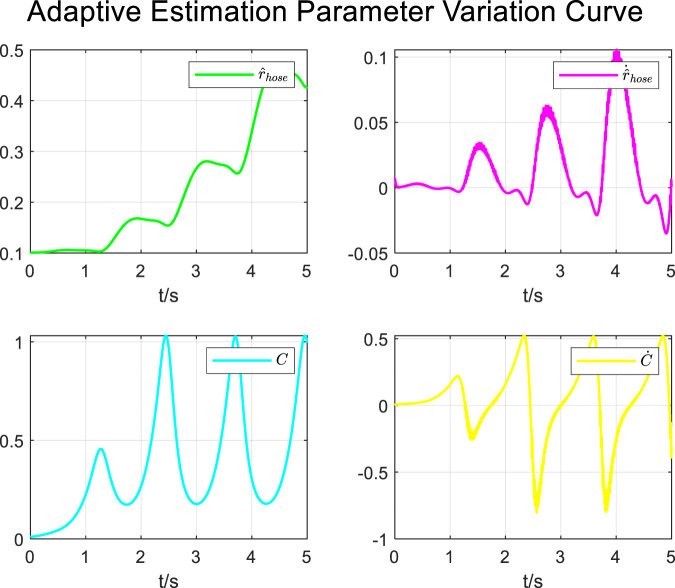
Adaptive estimation parameter variation curve for sinusoidal curve.

During the tracking process of airway pressure to a sinusoidal pressure signal under the adaptive control strategy, the results of the changes in the estimated parameters are shown in Figure 13.

It is evident that the adaptive estimation parameters converge within two–three expectoration cycles, meeting the practical clinical usage requirements.

**FIGURE 14:**
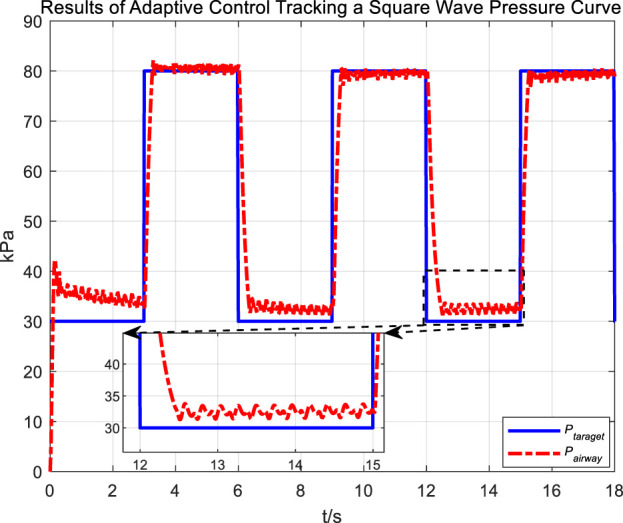
Results of adaptive control tracking a square wave pressure curve.

Figure 14 and 16 display the tracking performance of airway pressure to a square wave pressure signal under the adaptive control strategy.

**FIGURE 15:**
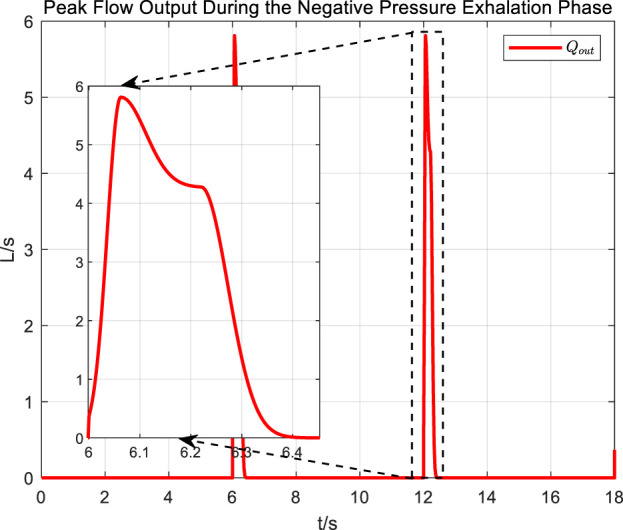
Peak flow output during the negative pressure exhalation phase of a square wave pressure curve.

The abrupt pressure changes of the square wave signal are consistent with the normal operating conditions of cough assist systems that generate negative pressure cough airflow. From the measurement results of cough airflow flow rates during the sudden drop phase of the square wave pressure signal in Figure 15, it is evident that the peak flow rate approaches 6 L/s, which is higher than the maximum expiratory flow rate of 1.1 L/s produced by a forceful natural cough. This demonstrates that under the adaptive control strategy, while regulating airway pressure, the cough assist system can still generate a forceful cough airflow.

**FIGURE 16:**
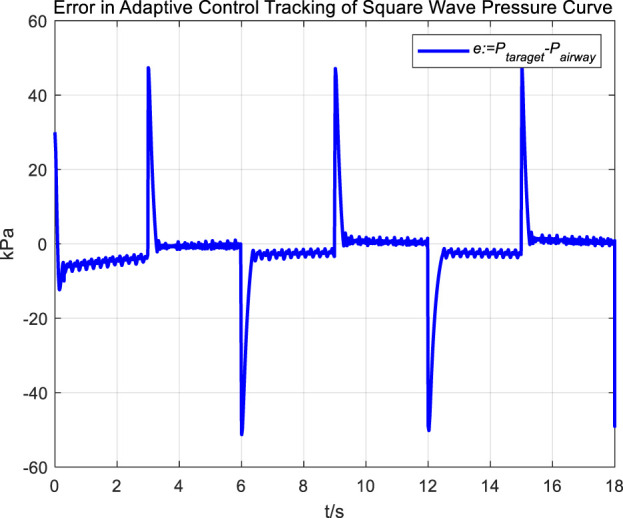
Error in adaptive control tracking of square wave pressure curve.

**FIGURE 17:**
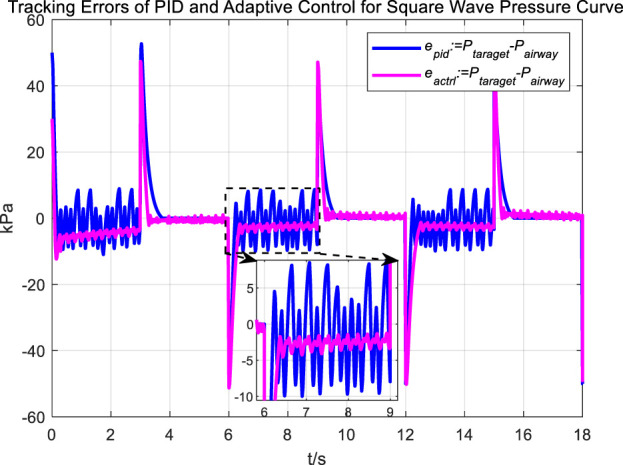
Tracking errors of PID and adaptive control for square wave pressure curve tracking.

Figures 16, 17 Provides a clear visual representation of the differences in the effectiveness of the adaptive control strategy and PID control in regulating airway pressure to track a square wave pressure signal.

Observations from the results under the square wave pressure curve lead to the same conclusions as those under the sinusoidal pressure curve. Under the adaptive control strategy, the regulation of airway pressure during the expectoration exhalation process has significantly improved. There is no overshoot, and although there is a steady-state error of approximately −3 kPa, oscillations have been reduced to ±1 kPa, which is a tenfold decrease.

**FIGURE 18:**
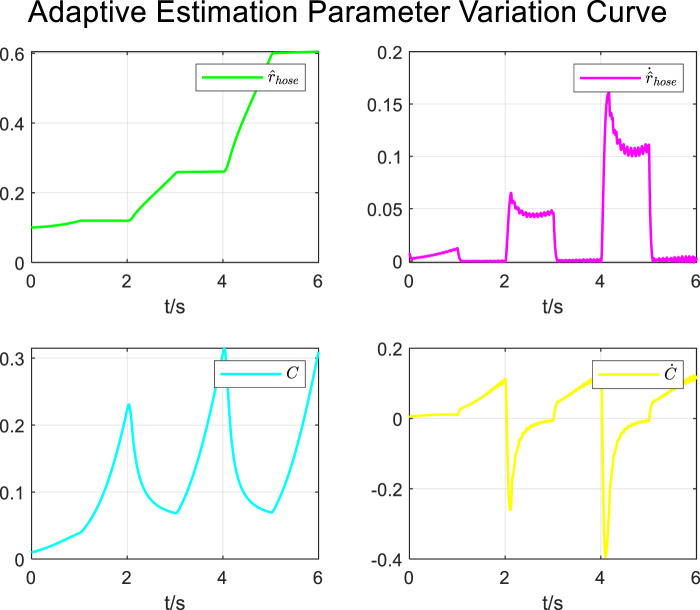
Adaptive estimation parameter variation curve for square wave curve.

During the tracking process of airway pressure to a square wave pressure signal under the adaptive control strategy, the results of changes in the estimated parameters are shown in Figure 18.

Similar to the results under the sinusoidal pressure curve, the adaptive estimation parameters converged within two–three expectoration cycles under the square wave pressure curve, satisfying the practical clinical usage requirements.

## Discussion

The experimental results reveal that while the PID control strategy can regulate airway pressure during the expectoration process, there is noticeable overshoot and oscillation in tracking the target pressure signal. In practical applications with patients using the cough assist machine, such overshoot and oscillation, compared to the natural coughing air pressure, can cause discomfort and potential airway damage to the patient.

In contrast, under the proposed adaptive control strategy, the tracking of the anticipated pressure during the expectoration process exhibits significantly reduced oscillations, greatly diminished overall tracking errors, and no overshoot in airway pressure. Furthermore, the experimental results indicate that under the adaptive control strategy, the estimated parameters can be updated and converge to stability within 5–6 s. In practical use of the cough assist machine, this can be achieved with just two to three coughing maneuvers, which meets the operational requirements of the scenario. This highlights the effectiveness of the adaptive control approach in achieving a more stable and accurate pressure management in cough assist systems.

In this study, we systematically compare the efficacy of PID control, a traditional control strategy, with the newly proposed adaptive control in managing airway pressure within an cough assist machine. PID control is widely used due to its broad applicability, straightforward parameter setting, and intuitive calculation process. However, a significant limitation of PID control is its fixed parameters, which lack flexibility in the face of the variability of patient physiological parameters typical in clinical environments. In our experiments, the PID control strategy led to pressure oscillations and overshoot, instabilities that could potentially cause unintended high airway pressures and additional physiological strain on patients, particularly for those with heightened sensitivity in their respiratory systems, such as patients with chronic obstructive pulmonary disease or asthma, highlighting the deficiencies of this control strategy.

In contrast, the adaptive control strategy, by dynamically adjusting control parameters to suit the current physiological state of the patient, demonstrated higher control precision and stability. The adaptive control system uses real-time feedback to optimize its performance, maintaining appropriate airway pressure under constantly changing clinical conditions, significantly reducing potential damage to the patient’s airways. Additionally, the adaptive control system reduces risks associated with improper operation of the equipment, such as when operators fail to adjust settings timely to match changes in patient status.

While the adaptive control strategy still has room for optimization, such as ensuring control effectiveness while maintaining algorithm efficiency and stability in emergency medical situations to avoid introducing additional computational delays, it is particularly crucial. Therefore, future research will consider developing more efficient adaptive algorithms to shorten response times, enhance adaptive control efficiency, further personalize treatment plans, predict and reduce potential adverse reactions, and ultimately increase the safety and effectiveness of therapy.

## Conclusion

MI-E cough assist system have not considered the impact of cough airflow pressure on therapeutic outcomes. Additionally, these devices lack effective solutions for controlling airway pressure during the expiratory phase, where significant negative pressure can lead to airway collapse.

This paper optimizes the design of cough assist machines and explores the application of PID and adaptive control methods in regulating airway pressure within these devices. Comparative experiments with PID controllers have revealed the significant advantages of the proposed adaptive control in reducing oscillations and overshoot, offering more stable and precise airway pressure regulation. Moreover, this adaptive control strategy does not impact the generation of peak flow rates necessary for effective mucus clearance. This capability has visible benefits in terms of clinical safety, which is crucial for enhancing patient outcomes and reducing the risk of complications.

In summary, this study presents an improved design for MI-E cough assist system. By employing adaptive control, the device effectively regulates airway pressure during the assisted coughing process facilitated by the cough assist system, providing a novel solution to enhance safety and prevent airway damage in critically ill patients with expectoration disorders. This approach promises to offer safer and more effective expectoration support for patients.

## Data Availability

The original contributions presented in the study are included in the article/Supplementary Material, further inquiries can be directed to the corresponding author.
